# The geometry of discrete asymptotic-geodesic 4-webs in isotropic 3-space

**DOI:** 10.1007/s00605-023-01916-0

**Published:** 2023-11-03

**Authors:** Christian Müller, Helmut Pottmann

**Affiliations:** 1 Institute for Discrete Mathematics and Geometry, TU Wien, Wiedner-Hauptstraße 8-10/104, 1040 Vienna, Austria; 2 Computer, Electrical and Mathematical Science and Engineering Division, KAUST, 23955-6900 Thuwal, Kingdom of Saudi Arabia

**Keywords:** Discrete webs, Isotropic minimal surfaces, Isotropic Voss-nets, Discrete differential geometry, 53A70, 53A60, 51N15, 53A10

## Abstract

The geometry of webs has been investigated over more than a century driven by still open problems. In our paper we contribute to extending the knowledge on webs from the perspective of the geometry of webs on surfaces in three dimensional space. Our study of AGAG-webs is motivated by architectural applications of gridshell structures where four families of manufactured curves on a curved surface are realizations of asymptotic lines and geodesic lines. We describe all discrete AGAG-webs in isotropic space and propose a method to construct them. Furthermore, we prove that some sub-nets of an AGAG-web are timelike minimal surfaces in Minkowski space and can be embedded into a one-parameter family of discrete isotropic Voss nets.

## Introduction

With the present paper we contribute to the ongoing development and investigation of the geometry of webs. The concept *geometry of webs* is so multifaceted that topics which fit under its roof reach from differential topology, differential geometry, algebraic geometry to discrete geometry.

A typical characterization of a *web* or an *n*-*web* consists of *n* families of curves on a surface or in the plane, such that, through each point, there is a curve of each family passing through that point, and such that any two curves from different families intersect each other at exactly one point (see, e.g., [1]). There exist of course generalizations of that definition in several directions.

The existence of families of curves on surfaces which fulfill the web condition is (at least locally) nothing particularly special. However, it becomes an interesting geometric or topological problem as we ask for conditions on the families of curves. For example the classification of all 3-webs in the plane consisting of three families of straight lines has been found by Graf and Sauer [2]: straight-lined 3-webs in the plane consist of all tangents of an algebraic curve of class 3. Remarkably, the generalization of this problem to the classification of all 3-webs in the plane where all curves are circles [1], is still an open problem.

The classical results on the geometry of webs which have mainly been developed by Blaschke and his school can be found in [1] and the survey article [3].

On curved surfaces in 3-space one can ask for webs where the foliating families of curves are determined partially or completely by the curvature and metric of the surface. For example Stephanidis [4] gives an integral condition for a negatively curved surface such that the families of curvature lines and asymptotic lines form a 4-web. Furthermore, it turns out that such 4-webs on surfaces with constant mean curvature exist only on minimal surfaces and surfaces of revolution.

Koch [5, 6] goes on with the investigation of 4-webs consisting of asymptotic lines and curvature lines (including topological aspects of the networks). It can be shown that such 4-webs exist if and only if the asymptotic net *f*(*u*, *v*) satisfies $\Vert f_u\Vert = \Vert f_v\Vert$. In [6] Koch works through well known surface classes and checks them for the existence of these special 4-webs or gives conditions for when they exist. For example they exist on all negatively curved surfaces of revolution, all non-developable ruled surfaces, all pseudospherical surfaces, all minimal surfaces, and all negatively curved parts of Dupin cyclides.

Apart from the many theoretical investigations of webs there are practical applications of webs in the areas of fabrication and architecture. Pottmann et al. [7] investigate gridshells fabricated by bending three families of long, thin, and rectangular strips of bendable materials (like wood or metal sheets) in the combinatorics of a discretized 3-web. In geometric terms such a web is a 3-web of geodesics on a curved surface. The bendable strips follow tangentially the shape of geodesics on the surface. Surfaces with 3-webs of geodesics have also been studied by Sauer [8] and Mayrhofer [9].

Another application actually was the motivation of our present paper. Schling et al. [10] investigate 4-webs on negatively curved surfaces in an architectural context. Thereby, two of the four families of curves are the asymptotic curves and the other two families consist of geodesics. The arrangement of curves around each point is such that they alternate cyclically (asymptotic- geodesic- asymptotic- geodesic). They therefore call those webs *AGAG-webs*.

In the present paper, we contribute to the theory of webs by extending our knowledge on AGAG-webs in a different Cayley-Klein geometry than in the above assumed Euclidean space, namely in isotropic geometry.

Simply put, *isotropic geometry* is the geometry of $\mathbb {R}^3$ together with the pseudometric (see, e.g., [11])

$$\begin{aligned} d((x_1, y_1, z_1), (x_2, y_2, z_2)) = \sqrt{(x_1 - x_2)^2 + (y_1 - y_2)^2}. \end{aligned}$$

Isotropic geometry has been developed by Strubecker [12–14] and can be looked up in the monograph of Sachs [11] and with a view towards applications in architecture in [15].

In isotropic space there is one direction (typically the *z*-axis direction) distinguished from the other directions. It therefore comes natural that surface parametrizations as graph *z*(*x*, *y*) over the *xy*-plane are also distinguished from general parametrizations. That fact leads to a natural appearance of isotropic geometry in the treatment of stress functions in mechanics [16]. Notions of stress and elasticity in mechanics appear as geometric properties of the Airy (stress) surface which are solutions to the biharmonic equation $\Delta \Delta z(x, y) = 0$.

Furthermore, we contribute to the understanding of isometries of surfaces in isotropic geometry, which haven’t been discussed so far, apparently because of the degeneracy of the metric. The notion of isometries of surfaces needed to be adapted to describe something sensible.

We develop our study of isotropic AGAG-webs in the setting of discrete differential geometry in the sense of Bobenko and Suris [17].

In Sect. 2 we give a short introduction into isotropic geometry and its related concepts from Cayley–Klein geometries that we need in our paper. We introduce different classes of discrete nets and their relations and important formulas in Sect. 3, before we geometrically describe all discrete AGAG-webs in isotropic space in Sect. 4 including a method of an explicit way to construct them. Finally, in Sect. 5 we examine various types of sub-nets of a discrete isotropic AGAG-web and interpret their geometric nature in isotropic, Minkowski, and dual Minkowski space. We encounter discrete timelike minimal surfaces, K-nets (discrete constant Gaussian curvature nets), Voss nets together with a newly developed notion of discrete isometry in isotropic space.

## Isotropic geometry and other Cayley–Klein geometries

The projective standard model of a *Cayley–Klein geometry* is a complex extended real *n*- dimensional projective space $P^n$ together with a group of transformations that leave an object *F*, the *absolute*, fixed [18]. This absolute consists of a collection of quadrics and can be real or imaginary. A straight line (real or imaginary) passing through the absolute is called *isotropic*. Throughout the paper we will encounter four Cayley–Klein geometries: Euclidean, isotropic, Minkowski and dual Minkowski.

**Fig. 1:**
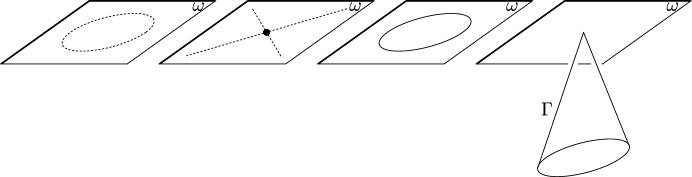
Illustration of Cayley–Klein-spaces with their absolutes. The ideal plane is denoted by $\omega$. *Left:* The absolute of Euclidean geometry is a regular imaginary conic in the ideal plane. *Second from left:* The absolute of isotropic geometry is a pair of complex conjugate straight lines in the ideal plane which intersect in a real point at infinity. *Third from left:* The absolute of Minkowski (or pseudo-Euclidean) geometry is a regular conic in the ideal plane. *Right:* The absolute of dual Minkowski (or dual pseudo-Euclidean) geometry is a cone $\Gamma$ (or better cylinder since its tip is a point at infinity)

Even though the Cayley–Klein spaces are defined in a projective invariant way we will use a standard coordinate system of homogeneous coordinates in which the absolutes assume simple and commonly used equations. The corresponding absolutes are:

$$\begin{aligned} \begin{array}{lll} \textrm{Euclidean geometry }&{} x_0 = 0,\ \ x_1^2 + x_2^2 + x_3^2 = 0 \\ \textrm{isotropic geometry }&{} x_0 = 0,\ \ (x_1 + i x_2) (x_1 - i x_2) = 0 \\ \textrm{Minkowski geometry }&{} x_0 = 0,\ \ x_1^2 + x_2^2 - x_3^2 = 0 \\ \mathrm{dual-Minkowski geometry} &{} x_1^2 + x_2^2 - x_3^2 = 0 \end{array} \end{aligned}$$

where $i = \sqrt{-1}$ is the imaginary unit. For an illustration see Fig. 1.

The main scene of the present paper is the *isotropic space* $I_3$. The absolute of isotropic geometry is a pair of complex conjugate lines in the ideal plane (plane at infinity) $\omega$ which intersect in a real point at infinity, the *absolute point*, representing the *(full) isotropic direction*.

Straight lines passing through the absolute point are called *isotropic straight lines* and planes passing through the absolute point are called *isotropic planes*.

With the above choice of the absolute, *orientation preserving isotropic isometries* are represented by affine transformations in $\mathbb {R}^3$ of the form

$$\begin{aligned} I_3 \longrightarrow I_3:\quad \begin{pmatrix}x\\ y\\ z\end{pmatrix} \longmapsto \begin{pmatrix} \cos \varphi &{}-\sin \varphi &{}0\\ \sin \varphi &{} \cos \varphi &{}0\\ c_1 &{} c_2 &{} 1 \end{pmatrix} \begin{pmatrix}x\\ y\\ z\end{pmatrix} + \begin{pmatrix}a\\ b\\ c\end{pmatrix}. \end{aligned}$$

The orthogonal projection $(x, y, z) \mapsto (x, y)$ into the plane $z = 0$ is called *top view*. Consequently, isotropic isometries are composed by Euclidean transformations in the top view and shear transformations in *z*-direction. Therefore the Euclidean distance of the top view of two points is an isotropic invariant of two points $p_i = (x_i, y_i, z_i)$ with $i = 1, 2$ and can therefore be denoted as *isotropic distance*:

$$\begin{aligned} d(p_1, p_2) = \sqrt{(x_1 - x_2)^2 + (y_1 - y_2)^2}. \end{aligned}$$

Two non-isotropic planes $\varepsilon _1, \varepsilon _2$ with equations $z = u_i x + v_i y + w_i$ with $i = 1, 2$ intersect in an *isotropic angle*

$$\begin{aligned} \psi (\varepsilon _1, \varepsilon _2) = \sqrt{(u_2 - u_1)^2 + (v_2 - v_1)^2}. \end{aligned}$$

A projective duality is an inclusion reversing bijective relation between a projective space $P^n$ and its dual space $P^{n*}$. In three-space the duality swaps points with planes but maps straight lines to straight lines. A speciality of isotropic geometry is the existence of a projective duality which additionally swaps metric quantities like the isotropic distance with the isotropic angle. This so called *metric duality* $\delta$ can be realized by a null system via

1 $$\begin{aligned} \delta : P^3 \longleftrightarrow P^{3*},\quad \text {point}\ (a, b, c) \longleftrightarrow \text {plane}\ b x - a y - z + c = 0, \end{aligned}$$

in affine coordinates. *Null system* means that corresponding points *p* and planes $\delta (p)$ in the duality are incident: $p \in \delta (p)$. Note that points with the same top view are mapped to parallel planes and vice versa. Important properties of metric duality are:

### Lemma 1

[11] In the metric duality $\delta$ two points with isotropic distance *d* are mapped to two planes with intersection angle *d* and vice versa:

$$\begin{aligned} d(p_1, p_2) = \psi (\delta (p_1), \delta (p_2)). \end{aligned}$$

### Lemma 2

The top view of a straight line *L* and its image under the metric duality $\delta (L)$ are parallel.

### Proof

Let *L* be spanned by two points $p = (p_1, p_2, p_3)$, $q = (q_1, q_2, q_3) \in \mathbb {R}^3$. The two planes $\delta (p), \delta (q)$ have normal vectors $(p_2, -p_1, -1)$ and $(q_2, -q_1, -1)$, respectively. The direction of the line of intersection of these two planes is parallel to the cross product of their normal vectors

$$\begin{aligned} \begin{pmatrix} p_2\\ -p_1\\ -1\end{pmatrix} \times \begin{pmatrix} q_2\\ -q_1\\ -1\end{pmatrix} = \begin{pmatrix}p_1 - q_1\\ p_2 - q_2\\ *\end{pmatrix} \end{aligned}$$

and therefore parallel to $p - q$ in the top view. $\square$

The paraboloid *S* with equation

2 $$\begin{aligned} z = \frac{1}{2} \left( x^2 +y^2\right) \end{aligned}$$

is treated as *isotropic unit sphere*. The isotropic Gauss map between an admissible surface and the isotropic unit sphere is realized through parallel tangent planes. *Admissible* surfaces in isotropic geometry are regular surfaces without isotropic tangent planes. The *isotropic Gaussian curvature*, which is also called *relative curvature* in the work of Strubecker [13], is the limit of the ratio of areas between the surface and its Gaussian image as the diameter of the area goes to zero.

Since distances are measured in the top view, a *geodesic* is a curve on an admissible surface whose top view is a straight line which makes the investigation of the geometry of geodesics in isotropic geometry simpler than in Euclidean geometry.

An often used notion of discrete Gauss image of polyhedral surfaces in Euclidean geometry is the dual net consisting of those points on the unit sphere where the tangent plane is parallel to the corresponding face on the given polyhedral surface. To obtain the *discrete isotropic Gauss image* we only replace the Euclidean unit sphere by the isotropic unit sphere *S*. A vertex star with central vertex *v* corresponds to a face $\sigma$ in the Gauss image.

We follow commonly used ideas (see, e.g., [19]) to define the *discrete isotropic Gauss curvature* as the ratio of the area enclosed by $\sigma$ divided by some weighted area of the vertex star around *v*:

3 $$\begin{aligned} K = \frac{\text {area}(\sigma )}{\text {weighted area}(v)}, \end{aligned}$$

where the weighted area of a vertex star can be the arithmetic mean or the sum of Voronoi cells of the adjacent face areas.

In both geometries, Euclidean as well as isotropic geometry, there is a relation between surfaces with constant Gaussian curvature and the existence of Chebyshev nets (i.e., nets *f* with $\Vert f_u\Vert = \Vert f_v\Vert = const$). Isotropic surfaces with constant Gaussian curvature are characterized by their asymptotic nets forming a Chebyshev net. *Discrete Chebyshev nets* are nets with quadrilateral faces where opposite edges have equal lengths. *Discrete isotropic Chebyshev nets* appear in the top view as translational nets where each face is a parallelogram.

Finally note, that there are also other duality mappings ${\tilde{\delta }}: P^3 \longleftrightarrow P^{3*}$ in $I^3$ which preserve the metric duality. For example the polarity in the paraboloid *S* with Eq. (2) which reads

4 $$\begin{aligned} {\tilde{\delta }}: P^3 \longleftrightarrow P^{3*},\quad (a, b, c) \longleftrightarrow a x + b y - z - c = 0. \end{aligned}$$

### Lemma 3

The top view of the discrete Gauss map coincides with the top view of the image under the polarity ${\tilde{\delta }}$.

### Proof

Consider a plane $\varepsilon$ with equation $a x + b y - z - c = 0$. Its image under the polarity is ${\tilde{\delta }}(\varepsilon ) = (a, b, c)$ with top view (*a*, *b*). On the other hand the point on *S* with the same top view (*a*, *b*) is $\left( a, b, \frac{1}{2}\left( a^2 + b^2\right) \right)$. The tangent plane in that point has normal vector $(a, b, -1)$ and is therefore parallel to the given plane. $\square$

## A-nets from planar Kœnigs nets

AGAG-webs contain an asymptotic sub-net (A-net) and a geodesic sub-net. A-nets then again are related to Kœnigs nets. Since the aim of this paper is to investigate discrete AGAG-webs we therefore start by recalling some facts about discrete A- and Kœnigs nets.

We investigate discrete nets which locally have $\mathbb {Z}^2$ combinatorics, i.e., a net is parametrized by $f: U \rightarrow \mathbb {R}^3$ over a simply connected domain $U \subset \mathbb {Z}^2$. A planar net is of the form $f: U \rightarrow \mathbb {R}^2$. We will often write parameters as indices: $f_{ij} = f(i, j)$, $f_{i + 1, j} = f(i + 1, j)$, etc. The discrete partial derivatives (forward differences) are denoted by

$$\begin{aligned} \delta _i f_{ij}:= f_{i + 1, j} - f_{ij} \quad \text {and}\quad \delta _j f_{ij} = f_{i, j + 1} - f_{ij}. \end{aligned}$$

**Fig. 2:**
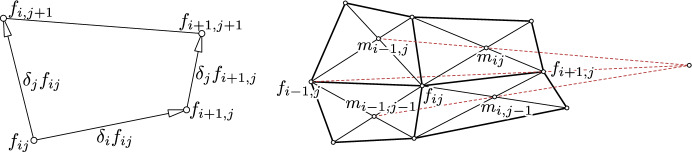
*Left:* Notation: parameter are denoted as indices and discrete partial derivatives by $\delta _i$, $\delta _j$. *Right:* Configuration of a discrete Kœnigs net. The vertices $m_{ij}$ are the intersection points of the diagonals. A net is a discrete Kœnigs net if and only if the three lines $\left( m_{ij} \vee m_{i - 1, j}\right)$, $\left( m_{i, j - 1} \vee m_{i - 1, j - 1}\right)$, $\left( f_{i + 1, j} \vee m_{i - 1, j}\right)$ intersect in a common point

See Fig. 2 (left) for an illustration of the notation.

The surface classes that we will work with the most are *Q-nets* (*conjugate nets*) which are nets where all faces are planar quadrilaterals, and *A-nets* (*asymptotic nets*) which are nets where each vertex star (which is a vertex with its four edge-connected neighbors) is planar (see e.g., [17]).

Both types of nets, Q-nets and A-nets, are preserved under projective transformations. Furthermore, both types of nets are preserved under projective dualities. Any duality $\delta$ maps the faces and vertices of a Q-net to the vertices and faces of its dual Q-net (see Fig. 9 right). Similarly, any duality $\delta$ maps planar vertex stars of A-nets to planar vertex stars its dual A-net.

Nets with $\mathbb {Z}^2$ combinatorics have two *diagonal nets* where the edges are diagonals of the $\mathbb {Z}^2$ lattice and where the faces correspond to every other vertex star of $\mathbb {Z}^2$. Thus, the vertices of one diagonal net are $\{(i, j) \in \mathbb {Z}^2 \mid i + j \equiv 0 \mod 2\}$ and of the other $\{(i, j) \in \mathbb {Z}^2 \mid i + j \equiv 1 \mod 2\}$. For an illustration of the combinatorics see the image in the appendix. The two diagonal nets of an A-net are Q-nets since the vertex stars of A-nets are planar per definition.

### Definition 1

Two nets $f, {\hat{f}}: \mathbb {Z}^2 \supset U \rightarrow \mathbb {R}^3$ are said to be *parallel* or *related by a discrete Combescure transformation*, if at each point corresponding partial derivative vectors are parallel, i.e., $\delta _i f_{ij} \parallel \delta _i {\hat{f}}_{ij}$ and $\delta _j f_{ij} \parallel \delta _j {\hat{f}}_{ij}$.

### Kœnigs nets

The faces of Q-nets are planar quadrilaterals which implies that the diagonals of each quadrilateral intersect in a point $m_{ij} = \left( f_{ij} \vee f_{i + 1, j + 1}\right) \cap \left( f_{i + 1, j} \vee f_{i, j + 1}\right)$. It is therefore possible to consider the ratio of diagonal segments $\left( f_{ij} - m_{ij}\right) : \left( f_{i + 1, j + 1} - m_{ij}\right)$ which is a ratio of parallel vectors.

The so called discrete Kœnigs nets form a subclass of Q-nets [17]. A Q-net $f: U \rightarrow \mathbb {R}^3$ is a *discrete Kœnigs net* if and only if there exists a real-valued function $\nu : U \rightarrow \mathbb {R}{\setminus }\{0\}$ such that for all faces

$$\begin{aligned} \frac{\nu _{i + 1, j + 1}}{\nu _{ij}} = \frac{f_{i + 1, j + 1} - m_{ij}}{f_{ij} - m_{ij}} \quad \text {and}\quad \frac{\nu _{i + 1, j}}{\nu _{i, j + 1}} = \frac{f_{i + 1, j} - m_{ij}}{f_{i, j + 1} - m_{ij}}. \end{aligned}$$

Kœnigs nets can be *dualized* in the sense that there exists a *dual* net ${\hat{f}}: U \rightarrow \mathbb {R}^3$ solving the system of difference equations (cf., [17, Th. 2.31])

5 $$\begin{aligned} \delta _i {\hat{f}}_{ij} = \frac{\delta _i f_{ij}}{\nu _{ij} \nu _{i + 1, j}} \quad \text {and}\quad \delta _j {\hat{f}}_{ij} = \frac{\delta _j f_{ij}}{\nu _{ij} \nu _{i, j + 1}}. \end{aligned}$$

The dual net ${\hat{f}}$ is a Kœnigs net as well with corresponding function ${\hat{\nu }} = \nu ^{-1}$.

#### Example 1

Consider a discrete translational *f* net which is generated by translating a polygon along another polygon. Each face of such a translational net is a parallelogram and the intersection points $m_{ij}$ are the midpoints of the diagonals. A translational net *f* together with the function $\nu _{ij}:= (-1)^i$ fulfills the above properties and is therefore a discrete Kœnigs net. Translational nets will play an important role in Sect. 5.

Another equivalent characterization of Kœnigs nets in terms of incidence geometry is the following. We will need this characterization later in the proof of Theorem 7.

#### Theorem 4

[Th. 3.10 [20]] A net is a discrete Kœnigs net if and only if the three lines $\left( m_{ij} \vee m_{i - 1, j}\right)$, $\left( m_{i, j - 1} \vee m_{i - 1, j - 1}\right)$, $\left( f_{i + 1, j} \vee f_{i - 1, j}\right)$ intersect in a common point. For an illustration see Fig. 2 (right).

Let us consider a Kœnigs net in the plane $f = \left( f^1, f^2\right) : U \rightarrow \mathbb {R}^2$. Its *Moutard lift* $n: U \rightarrow \mathbb {R}^3$ with

6 $$\begin{aligned} n:= \nu ^{-1} \left( f^1, f^2, 1\right) \end{aligned}$$

is a *discrete T-net* fulfilling the so called *discrete Moutard equation with minus sign* (cf., [17, Th. 2.32])

$$\begin{aligned} n_{i + 1, j + 1} - n_{ij} = \textstyle \frac{\nu _{i + 1, j + 1}^{-1} - \nu _{ij}^{-1}}{\nu _{i, j + 1}^{-1} - \nu _{i + 1, j}^{-1}} \left( n_{i, j + 1} - n_{i + 1, j}\right) . \end{aligned}$$

Such T-nets will appear later as special normal vector fields of A-nets (Theorem 5).

### A-nets

Each vertex star of an *A-net* is planar. Consequently, there is a normal vector *n* at each vertex which is orthogonal to each adjacent edge

$$\begin{aligned} n_{ij} \perp \delta _i f_{ij},\, \delta _j f_{ij},\, \delta _i f_{i - 1, j},\, \delta _j f_{i, j - 1}. \end{aligned}$$

Such a discrete normalized normal vector field does not define an A-net uniquely however the normal vectors equipped with a particular choice of individual lengths for each normal vector can determine an A-net uniquely: A-nets *f* in $\mathbb {R}^3$ (up to translation) are in a one-to-one correspondence with T-nets *n* via the *discrete Lelieuvre representation* (cf., [17, Th. 2.43])

7 $$\begin{aligned} \delta _i f_{ij} = n_{i + 1, j} \times n_{ij}, \quad \text {and}\quad \delta _j f_{ij} = n_{i, j + 1} \times n_{ij}, \end{aligned}$$

where “$\times$” denotes the vector cross product in $\mathbb {R}^3$. As we put together the above steps we conclude the following theorem which we will use later in the construction of an A-net.

#### Theorem 5

Let $f: U \rightarrow \mathbb {R}^2$ be a Kœnigs net in the plane. Its Moutard lift (6)

$$\begin{aligned} n = \nu ^{-1} \left( f^1, f^2, 1\right) \end{aligned}$$

is a discrete Lelieuvre representation of a discrete A-net.

### Projection of A-nets

The central projection of a smooth A-net to a plane is a smooth Kœnigs net in that plane. A proof of that can be found in [21] where smooth Kœnigs nets are called nets with equal invariants. Also a form of converse statement is proved in [21] which says that to each smooth Kœnigs net in the plane there exists a non-trivial smooth A-net in space which can be projected to the given smooth Kœnigs net. These properties also hold in the discrete setting. For example the fact that discrete A-nets are projected to discrete Kœnigs nets can be found as an Exercise in [17, Ex. 2.29]. We could not find any references for the converse result in the discrete setting, however, since we will need it later we will give an elementary proof here.

#### Theorem 6

Let $f: U \rightarrow \mathbb {R}^2$ be a discrete Kœnigs net in the plane with corresponding function $\nu : U \rightarrow \mathbb {R}\setminus \{0\}$ and let ${\hat{f}} = \left( {\hat{f}}^1, {\hat{f}}^2\right)$ denote its Kœnigs dual with corresponding function ${\hat{\nu }} = \nu ^{-1}$. Further, we rotate ${\hat{f}}$ by $\pi /2$ to obtain $\left( -{\hat{f}}^2, {\hat{f}}^1\right)$. Then the Moutard lift (6)

$$\begin{aligned} n:= \nu \left( -{\hat{f}}^2, {\hat{f}}^1, 1\right) \end{aligned}$$

of the rotated net ${\hat{f}}$ is the Lelieuvre representation of an A-net whose top view (horizontal projection) is the given net *f* up to translation. Note that the position vectors of the vertices of the Lelieuvre representation *n* are parallel to the normal vectors of the metric dual (1) of ${\hat{f}}$.

#### Proof

Theorem 5 implies that *n* is the Lelieuvre representation of an A-net. We only have to show that its top view is *f*.

**Fig. 3:**
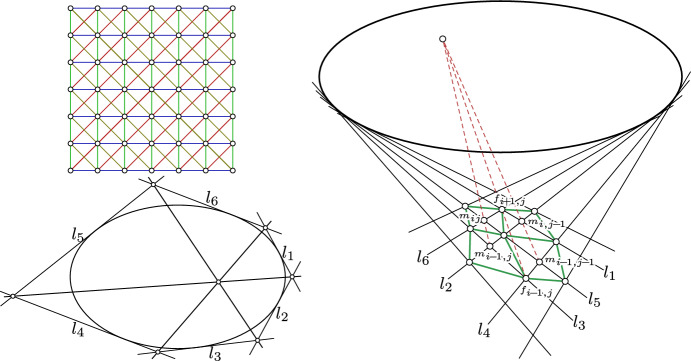
*Top-left:* The combinatorics of a 4-web consisting of four families of curves. Through each point there is a curve of each family passing through. Any three of the families of curves from the 4-web form a 3-web. *Bottom-left:* Brianchon’s theorem. *Right:* A $2 \times 2$ quadrilateral net *f* (green) in the plane with straight-lined diagonals $l_1, \ldots , l_6$. The six straight lines $l_1, \ldots , l_6$ envelope a conic if and only if *f* is a Kœnigs net. The incidence geometric Kœnigs property from Theorem 4 (see Fig. 2 right; here illustrated by dashed red lines) is a consequence of Brianchon’s theorem

We compute edge vectors of the A-net via the Lelieuvre representation (7)

$$\begin{aligned} n_{i + 1, j} \times n_{ij}= & {} \nu _{i + 1, j} \begin{pmatrix}-{\hat{f}}_{i + 1, j}^2\\ {\hat{f}}_{i + 1, j}^1\\ 1\end{pmatrix} \times \nu _{ij} \begin{pmatrix}-{\hat{f}}_{ij}^2\\ {\hat{f}}_{ij}^1\\ 1\end{pmatrix}\\= & {} \nu _{i + 1, j} \nu _{ij} \begin{pmatrix}{\hat{f}}_{i + 1, j}^1 - {\hat{f}}_{ij}^1\\ {\hat{f}}_{i + 1, j}^2 - {\hat{f}}_{ij}^2\\ * \end{pmatrix} \overset{(5)}{=} \begin{pmatrix}\delta _i f_{ij}^1\\ \delta _i f_{ij}^2\\ *\end{pmatrix}. \end{aligned}$$

Consequently, we obtain the top view of this A-net by omitting the third component which therefore is the top view of $\delta _i f_{ij}$. The same holds for the other direction. $\square$

## Discrete webs from asymptotic and geodesic lines in isotropic geometry

In this section we derive a sensible notion of discrete isotropic AGAG-webs and give a full geometric characterization of these webs. We investigate properties of sub-nets of discrete isotropic AGAG-webs and describe a method of constructing them.

### AGAG-webs

An *n*-*web* consists of *n* families of curves on a surface or in the plane, such that, through each point, there is a curve of each family passing through that point, and such that any two curves from different families intersect each other at exactly one point (see, e.g., [1]). A very special 4-web in $\mathbb {R}^2$ is illustrated in Fig. 3 (top-left) consisting of all lines of the form $x = d_1$, $y = d_2$, $x + y = d_3$, $x - y = d_4$, with $d_1, \ldots , d_4 \in \mathbb {R}$.

Certainly, we can map such a special planar *n*-web (like the 4-web from above) to any embedded surface patch. Thus, the existence of an *n*-web on a surface by itself is not particularly worth noticing. However, it becomes significantly more interesting if such a web is determined by the geometry of the surface or of the geometric properties of the curves (for example webs of geodesics [7, 9]).

#### Example 2

In isotropic geometry all 3-webs of geodesics on an admissible surface (i.e., without isotropic tangent planes) are obtained by projecting a straight-lined 3-web from the *xy*-plane to the surface. Straight-lined 3-webs in the plane have been fully classified and consist of all tangents of an algebraic curve of class 3 (see, e.g., [1]).

An application is the motivation for the present paper. Schling et al. [10] investigate 4-webs on negatively curved surfaces in an architectural context. Thereby, two of the four families of curves are the asymptotic curves and the other two families consist of geodesics. The arrangement of curves around each point is such that they alternate cyclically (asymptotic- geodesic- asymptotic- geodesic). We therefore recall formally the following definition.

#### Definition 2

[10] We call a 4-web on a negatively curved surface an *AGAG-web* if it consists of the two families of asymptotic curves and two families of geodesics such that the four curves through each point alternate cyclically (asymptotic- geodesic- asymptotic- geodesic).

Note that not every negatively curved surface carries an AGAG-web. The discretization of AGAG-webs is straightforward once we declare the notion of a discrete geodesic in $I^3$. We follow in our straightforward discretization the smooth characterization of geodesics in isotropic space.

#### Definition 3

A polygonal parameter curve on a discrete net is called a *discrete geodesic in isotropic space* $I^3$ if its top view is a straight line.

#### Definition 4

**Fig. 4:**
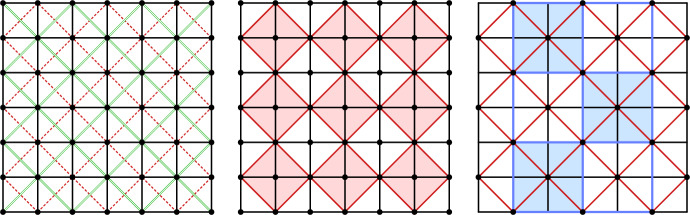
*Left:* The combinatorics of a discrete AGAG-web is a 4-web with vertices on the $\mathbb {Z}^2$ lattice such that the geodesic lines correspond to the vertical and horizontal parameter lines (black) and where its two diagonal nets form two A-nets (red dashed and green). *Center:* The combinatorics of a *relaxed* discrete AGAG-web where only one of the two diagonal nets of the net of geodesics is an A-net (red). For that relaxed setting we can show in Remark 1 the existence of a counter example to Theorem 10. *Right:* The blue net is one of the two diagonal nets of the red A-net

A *discrete AGAG-web* is a discrete 4-web with $\mathbb {Z}^2$ combinatorics such that the geodesic lines correspond to the vertical and horizontal parameter lines (black lines in Fig. 4 left) and where its two diagonal nets form two A-nets (red dashed and green nets in Fig. 4 left). A *discrete isotropic AGAG-web* is a discrete AGAG-web in isotropic geometry.

The vertices of the net of geodesics are $\mathbb {Z}^2$ whereas the vertices of the *A*-nets are $\{(i, j) \in \mathbb {Z}^2 \mid i + j \equiv 0 \mod 2\}$ and $\{(i, j) \in \mathbb {Z}^2 \mid i + j \equiv 1 \mod 2\}$, respectively.

#### Theorem 7

The top view of a discrete AGAG-web in isotropic geometry is a 4-web consisting of two discrete Kœnigs nets whose both families of diagonal curves are straight lines.

#### Proof

The top view of a discrete A-net is a discrete Kœnigs net and the top view of geodesic curves are straight lines. $\square$

#### Lemma 8

Consider a $2 \times 2$ quadrilateral net *f* in the plane with straight-lined diagonals $l_1, \ldots , l_6$ (as illustrated by Fig. 3 right). Then *f* is a Kœnigs net if and only if the six straight lines $l_1, \ldots , l_6$ envelope a conic.

#### Proof

According to the incidence geometric characterization of Kœnigs nets, *f* is a Kœnigs net if and only if the diagonal intersection points $m_{ij}, m_{i, j - 1}, m_{i - 1, j}, m_{i - 1, j - 1}$ together with $f_{i, j - 1}, f_{i, j + 1}$ fulfill the incidence relation described in Theorem 4 and illustrated by Fig. 2 (right). Consequently, *f* is a Kœnigs net if and only if the three lines $\left( m_{ij} \vee m_{i - 1, j}\right)$, $\left( m_{i, j - 1} \vee m_{i - 1, j - 1}\right)$, $\left( f_{i + 1, j} \vee f_{i - 1, j}\right)$ intersect in a common point. By Brianchon’s theorem (see e.g., [22] and Fig. 3 bottom-left) these three lines meet in a point if and only if the six lines

$$\begin{aligned}&l_1 := f_{i + 1, j} \vee m_{i, j - 1}{} & {} l_4 := f_{i - 1, j} \vee m_{i - 1, j - 1} \\&l_2 := m_{i - 1, j} \vee m_{i, j - 1}{} & {} l_5 := m_{i - 1, j - 1} \vee m_{ij} \\&l_3 := m_{i - 1, j} \vee f_{i - 1, j}{} & {} l_6 := m_{ij} \vee f_{i + 1, j} \end{aligned}$$

are in tangential contact with a conic. Consequently, the diagonal net of a net of straight lines enveloping a conic is a Kœnigs net. $\square$

#### Corollary 9

Two families of straight lines enveloping a conic constitute the diagonal net of a Kœnigs net.

#### Theorem 10

The geodesics in the top view of a discrete isotropic AGAG-web are straight lines enveloping a single conic.

#### Proof

**Fig. 5:**
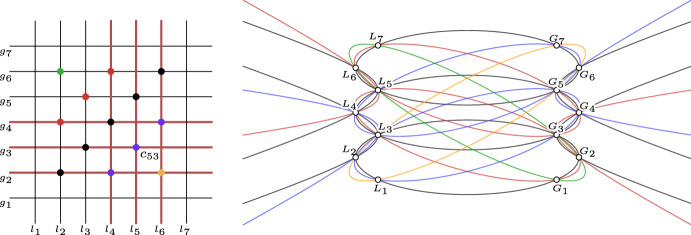
*Left:* Combinatorial illustration of the straight-lined diagonals of a Kœnigs net. Lemma 8 implies that the six lines $l_1, l_2, l_3, g_1, g_2, g_3$ are in tangential contact with a conic which is combinatorially illustrated by the dot at the intersection of $l_2$ with $g_2$. *Right:* A counterexample to the converse of Corollary 9 in the dual projective plane. Instead of straight lines tangent to conics we have points lying on conics. The points $L_1, \ldots$ and $G_1, \ldots$ lie on the $\mathbb {Z}^2$ lattice. The metric symmetry of the arrangement implies that the conics pass through the indicated points

The geodesics in the top view appear as straight-lined diagonals of two Kœnigs nets. Let us choose arbitrary coordinates $(i, j) \in \mathbb {Z}^2$. Lemma 8 implies that the six straight lines in the top view consisting of the three geodesics represented by the three combinatorially vertical lines through $(i - 1, j), (i, j), (i + 1, j)$ as well as the three combinatorially horizontal lines through $(i, j - 1), (i, j), (i, j + 1)$ envelope a conic $c_{ij}$. A combinatorial illustration with the six tangents corresponding to conic $c_{53}$ can be found in Fig. 5 (left). This holds for any (*i*, *j*). Since two “neighboring” conics $c_{ij}$ and $c_{i + 1, j}$ share five tangents (three horizontal and two vertical) they must be identical ($c_{ij} = c_{i + 1, j}$) as a conic is uniquely determined by five tangents [22]. Consequently, by induction all these conics are identical. $\square$

#### Remark 1

Theorem 10 would not be true if we relaxed the definition of a discrete AGAG-web by requiring that only one of the two diagonal nets of the geodesics net is an A-net (see Fig. 4 right). The common conic would no longer exist. A counterexample for what we claim here would consist of two collections of lines $l_1, \ldots , l_n$ and $g_1, \ldots , g_m$ (with *n*, *m* arbitrarily large) such that for any *i*, *j* with $i + j \equiv 0 \mod 2$ the six lines

$$\begin{aligned} l_{i - 1}, l_i, l_{i + 1}, g_{j - 1}, g_j, g_{j + 1} \end{aligned}$$

are in tangential contact with a conic and that there are at least two different such conics. See Fig. 5 (left) for a combinatorial illustration. We present such a counterexample in the dual projective plane which seems to be easier to visualize. For that, we construct two collections of points $L_1, \ldots , L_n$ and $G_1, \ldots , G_m$ (with *n*, *m* arbitrarily large) such that for any *i*, *j* with $i + j \equiv 0 \mod 2$ the six points

8 $$\begin{aligned} L_{i - 1}, L_i, L_{i + 1}, G_{j - 1}, G_j, G_{j + 1} \end{aligned}$$

are contained in a conic and that there are at least two different such conics. See Fig. 5 (right) for such a counterexample: $L_i = \left( (-1)^{i + 1}, 2 i - c\right)$ and $G_j = \left( (-1)^j, 2 j + c\right)$ (with an arbitrary constant *c*). Due to the symmetry of these points there exists a conic through any of the six points of the form (8).

It remains an open question if this type of counterexample and all its metric and projective equivalent versions is the only counterexample to the above described relaxed version of Theorem 10.

### Construction of discrete isotropic AGAG-webs

We would like to sketch a practical way to construct discrete isotropic AGAG-webs. In order to do that we start with its top view. By Theorem 10 we must assume the top view of the geodesic curves being straight lines enveloping a conic. We therefore choose two discrete one-parameter families of tangents of an arbitrarily chosen conic in the *xy*-plane.

**Fig. 6:**
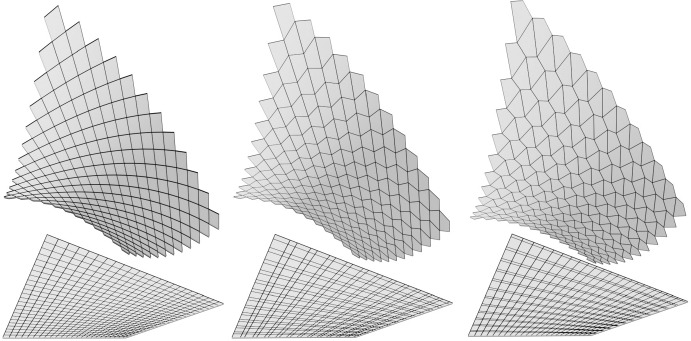
Simple Examples. The parameter curves of the nets on the bottom are tangent lines of a circle. The difference between the three figures is only the “speed” of the tangent lines, i.e., their arrangement. The nets above them are A-nets. The top views of the diagonal nets of these A-nets are the nets in the bottom row

**Fig. 7:**
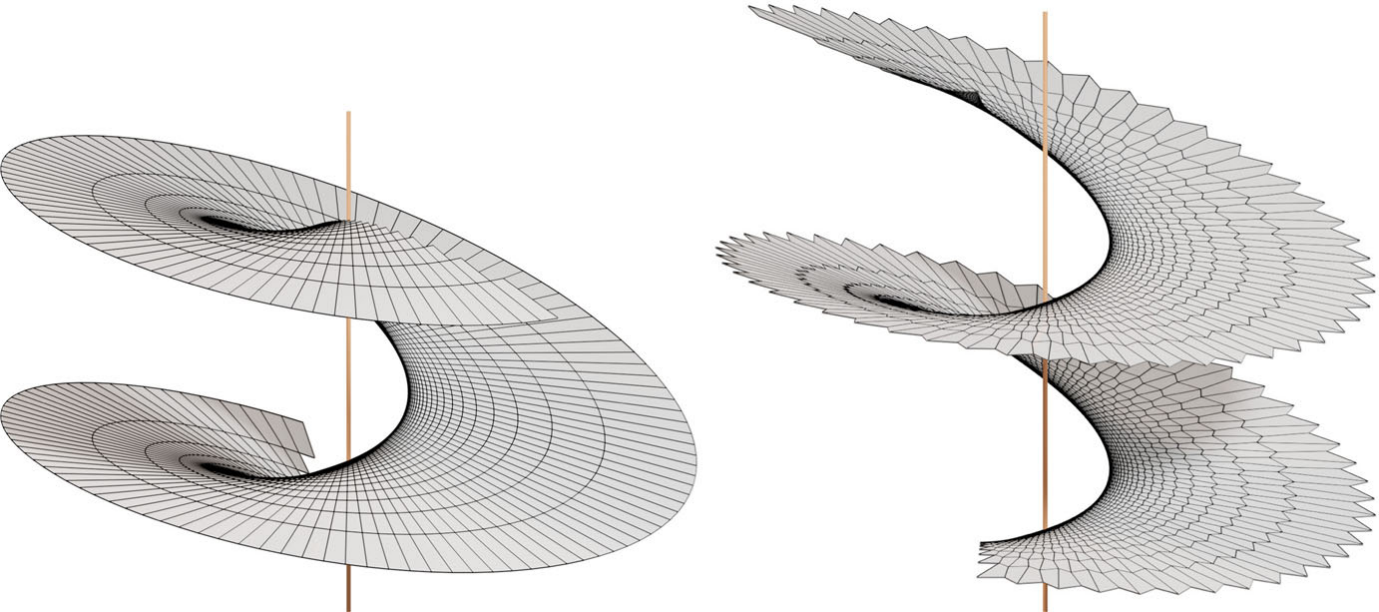
Discrete A-nets of two discrete AGAG-webs. The vertical line indicates the isotropic direction. The corresponding net of geodesics envelope a circle at constant speed: regular *n*-gon *left* and perturbed regular *n*-gon (*right*)

**Fig. 8:**
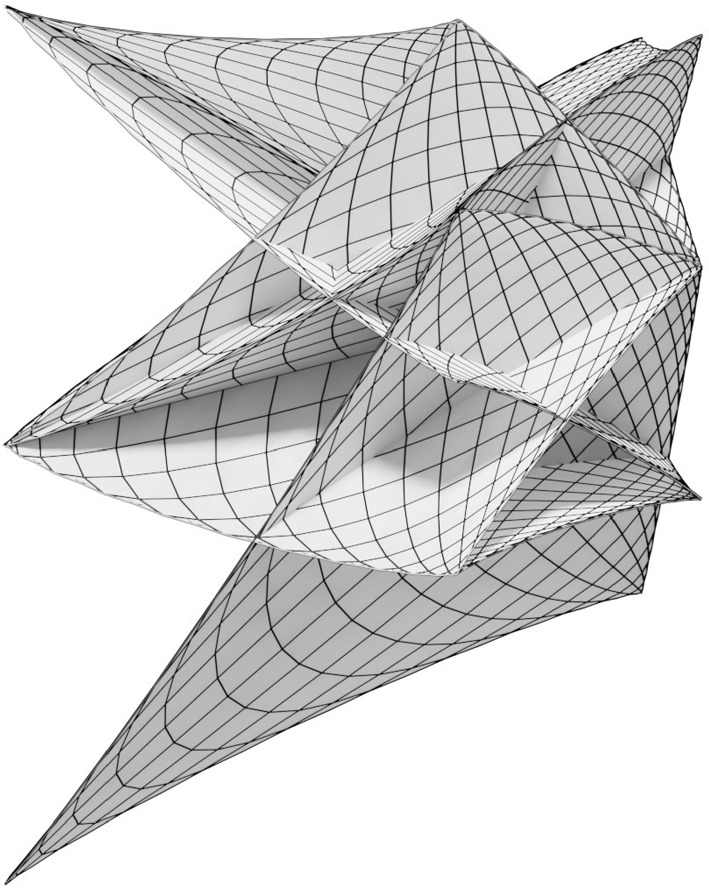
The tangents of the unit circle can be represented as $T(\varphi ):= (\cos \varphi , \sin \varphi ) + \mathbb {R}(-\sin \varphi , \cos \varphi )$. For the illustrated A-net of an AGAG-web we start with a net of two families of tangents to the unit circle parametrized by $T(\varphi _i)$, $T(\varphi _j)$, $i, j = 0, \ldots , 146$ with $\varphi _i =.3 \cos (i * \delta \varphi )$, $\varphi _j = \frac{\pi }{2} +.3 \cos (j * \delta \varphi )$, where $\delta \varphi = \frac{6 \pi }{146}$. Due to the $\cos$ function changing signs the tangents move back and forth and cover the same domain three times. This results in sharp edges on the A-net

By Corollary 9 its two diagonal nets are two planar Kœnigs nets. With the construction of Theorem 6 we obtain two A-nets whose top view coincide with these Kœnigs nets and generate the AGAG-webs. See Figs. 6, 7 and 8 for some examples.

The A-net in Fig. 7 lies in an affinely sheared helicoid. The two families of geodesics envelope in the top view a circle with the same constant angular velocity. Therefore one diagonal in each quad is a diameter of that circle and the other diagonal is orthogonal to it. In the smooth counterpart this corresponds to a surface whose asymptotic curves appear in the top view as concentric circles and their diameter lines. It follows immediately that one family of asymptotic curves is straight and thus the surface is a ruled surface. Due to the right isotropic angle between asymptotic directions we have a ruled isotropic minimal surface which is easily seen to be an isotropic helicoid (affine to a Euclidean helicoid).

## Discrete AGAG-webs, discrete minimal surfaces and voss nets

In the following we study the geometry of discrete isotropic AGAG-webs. As the geodesics appear in the top view as straight lines enveloping a conic, the planes carrying these geodesics are tangent to a quadratic cylinder $\Gamma$ with rulings that are isotropic lines in $I^3$, i.e., real rulings parallel to the *z*-axis. Most Q-nets in the following are formed by geodesic polylines in $I^3$. They are special cases of Q-nets with planar parameter lines, which have been studied recently in connection with multi-nets [23] and applications in architecture [24].

### Relation to timelike minimal surfaces in Minkowski space

Minimal surfaces are typically defined as surfaces with vanishing mean curvature *H* with an appropriate mean curvature notion in the respective geometry. On the other hand minimal surfaces can be seen as translation surfaces of two isotropic curves (see, e.g., [25] for Minkowski minimal surfaces) which generalizes Lie’s original generation of Euclidean minimal surfaces from two complex conjugate isotropic curves [26]. Discrete translation surfaces generated from discrete isotropic curves appear as timelike minimal surfaces in Minkowski space in [27].

Let us consider a discrete AGAG-web. By Theorem 10 there exists a conic which is enveloped by the straight lines corresponding to the top view of the geodesics of the discrete AGAG-web. We denote the quadratic cylinder whose top view coincides with this conic by $\Gamma$.

Let us consider this quadratic cylinder $\Gamma$ as the absolute of a Cayley-Klein geometry, namely a dual Minkowski geometry (cf. Sect. 2 and Fig. 1 right).

**Fig. 9:**
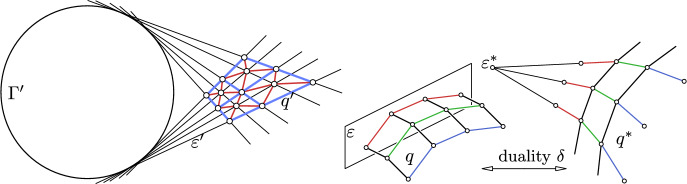
*Left:* Top view $\Gamma '$ of the cylinder $\Gamma$ and a discrete isotropic AGAG-web. The projection of one of the two A-nets is indicated in red and one of its diagonal nets *q* which projects to $q'$. The polygons of *q* lie in tangent planes $\varepsilon$ of $\Gamma$. *Right:* A projective duality $\delta$ maps a Q-net *q* to a Q-net $q^*$. Co-planar edges of a parameter polygon (contained in a plane $\varepsilon$) are mapped to edges meeting in a point $\varepsilon ^* = \delta (\varepsilon )$

#### Theorem 11

A diagonal net of an A-net of a discrete isotropic AGAG-web represents a timelike minimal surface in a dual Minkowski space, whose absolute is given by the cylinder $\Gamma$. The tangent planes of $\Gamma$ contain the geodesics of the web.

#### Proof

Let us consider one of the two diagonal nets of one of the two A-nets of a discrete AGAG-web. Such a diagonal net *q* is a Q-net because every diagonal net of an A-net is a Q-net. The discrete parameter lines of this Q-net *q* lie in tangent planes $\varepsilon$ of $\Gamma$ (see Fig. 9 left). Since dual Minkowski geometry is harder to visualize than the more familiar Minkowski geometry, we first apply a projective duality $\delta$ which maps the tangent planes of $\Gamma$ to the points of some conic *c* in the ideal plane $\omega$ $(x_0 = 0)$. We can assume that *c* agrees with the ideal conic

$$\begin{aligned} x_0 = 0, \quad x_1^2 + x_2^2 - x_3^2 = 0 \end{aligned}$$

(see Fig. 1 third from left). It is then the absolute of the Minkowski space based on the standard Minkowski scalar product. The projective duality $\delta$ maps the Q-net *q* to a Q-net $q^*$ (see Fig. 9 right for an illustration). The edges of the parameter lines of *q* are contained in tangent planes $\varepsilon$ of $\Gamma$. Such a planar parameter polygon gets mapped to the edges connecting two adjacent parameter lines of $q^*$. All these transversal edges are parallel, since they contain the ideal image point $\varepsilon ^*:= \delta (\varepsilon ) \in c$. This shows that all straight lines carrying the edges of $q^*$ meet *c* and thus are isotropic lines in Minkowski space.

Consequently, each face of $q^*$ is a parallelogram and thus the net $q^*$ is a translational net, generated by translating an isotropic polyline along another isotropic polyline. This generation is the Minkowski-counterpart to Lie’s generation of Euclidean minimal surfaces as translation surfaces (generated by conjugate complex minimal curves), and according to the work of Yasumoto [27] a characterization of timelike minimal surfaces in Minkowski space. $\square$

Note that there are two A-nets contained in a discrete AGAG-web each of which carrying two diagonal nets (see also the table in the appendix). Consequently there are four discrete timelike minimal surfaces contained in a discrete AGAG-web.

It has to be noted that discrete isotropic AGAG-webs are only related to special minimal surfaces in Minkowski space. This follows already from the fact that in the present setting we have negatively curved surfaces (represented by A-nets), while timelike minimal surfaces in Minkowski space may also be positively curved. This follows from their generation as translation surfaces.

### Isotropic Voss nets

A Q-net is called *conjugate* if the second fundamental form is diagonal, or equivalently, if the tangent vectors are orthogonal with respect to the second fundamental form. Surfaces which possess a conjugate net of Euclidean geodesics have been first studied by Voss [28] and are now called *Voss surfaces* or *Voss nets* or *V-nets*. Remarkably, they allow for isometric deformations under which the geodesic net remains conjugate. Discrete versions are flexible Q-nets: considering their planar faces as rigid bodies and their edges as hinges, they are mechanisms which perform one-parameter motions [8]. All flexible Q-nets have been classified by Izmestiev [29].

In what follows the notion of reciprocal-parallel nets will appear frequently. Two nets are called *combinatorially dual* to each other if the vertices of one net correspond to the faces of the other such that there is a natural bijection of the edges (edge-adjacent faces correspond to edge-adjacent vertices). We call two nets *reciprocal-parallel* to each other if they are combinatorially dual and if corresponding edges are parallel. Lemma 2 implies:

#### Lemma 12

Let *q* be a Q-net and $\delta$ the metric duality (1). Then the top views of corresponding edges of *q* and $\delta (q)$ are parallel to each other. They form a pair of reciprocal-parallel nets (so called reciprocal diagrams in the sense of graphic statics). The edge vectors of $\delta (q)'$ are force vectors which act in the corresponding edges of $q'$ to obtain equilibrium. The role of the two diagrams can be exchanged. One is the force diagram of the other.

It is also known that discrete V-nets are reciprocal-parallel to discrete K-nets (surfaces of constant Gaussian curvature [30–32]). In the language of graphic statics, they are reciprocal force diagrams of each other.

A discrete isotropic AGAG-web contains four Q-nets formed by geodesics. In analog to the Euclidean geometry Q-nets with geodesics as parameterlines in isotropic geometry can be considered *isotropic V-nets*.

#### Theorem 13

A diagonal net of an A-net *q* of a discrete isotropic AGAG-web is an isotropic V-net with the following properties:

(i) The net *q* is metrically dual to a translational net $q^*$ all whose edges are parallel to a quadratic cone.
(ii) The isotropic dihedral angle along each parameter line is constant.
(iii) The net *q* is reciprocal-parallel to an isotropic K-net *k*, i.e., a discrete surface with constant negative isotropic Gaussian curvature.

#### Proof

We start with (i): We apply the isotropic metric duality $\delta$, realized by the null system (1) that maps a point (*a*, *b*, *c*) to the incident plane $b x - a y - z + c = 0$. With the same arguments as in the proof of Theorem 11, the metric dual $q^*$ of a discrete isotropic V-net *q* is a translational net.

The parameter lines of *q* are tangent to the isotropic cylinder $\Gamma$ with equation

$$\begin{aligned} x_1^2 + x_2^2 - x_3^2 = 0. \end{aligned}$$

This cylinder $\Gamma$ is mapped by the duality to the conic with equation

$$\begin{aligned} x_0 = 0,\ \ x_1^2 + x_2^2 - x_3^2 = 0 \end{aligned}$$

in the ideal plane. Thus all edges of $q^*$ pass through that conic in the ideal plane and are therefore parallel to a quadratic cone through that ideal conic.

As for (ii), since $q^*$ is a translational net also its top view $q^*{}'$ is a translational net. Therefore, the edges connecting two neighboring parameter curves of $q^*$ are congruent, both in Euclidean and isotropic geometry. By the metric duality (Lemma 1), the isotropic dihedral angle of the isotropic V-net *q* is constant along each (planar) parameter line.

To show (iii) we first recall that $q^*$ is a translational net and therefore so is $q^*{}'$. Consequently, Example 1 implies that $q^*{}'$ is a Kœnigs net. Theorem 6 implies the existence of an A-net $k^*$ whose top view is $q^*{}'$. The metric dual *k* of $k^*$ is an A-net. Since vertices with the same top view, like $k^*$ and $q^*$, correspond to parallel planes in the metric duality, the planes carrying the vertex stars of $k^*$ are parallel to their corresponding faces in *q*. Furthermore, since two pairs of parallel planes intersect in parallel lines, corresponding edges in *q* and *k* must be parallel. Consequently, *q* and *k* are reciprocal-parallel.

Lemma 2 implies that the top view of corresponding edges of *k* and $k^*$ are parallel. Since $k^*{}' = q^*{}'$ is a translational net, parallelity of corresponding edges implies that $k'$ is a translational net. Since the top view of the A-net *k* is translational it is also an isotropic Chebyshev net.

Thus, *k* is a discrete version of a surface with an asymptotic Chebyshev net, i.e., a surface with constant negative isotropic Gaussian curvature [13]. $\square$

Basically all properties of isotropic K-nets known from the smooth setting can be transferred to the discrete case. This is particularly simple in the case where *k* is an isotropic rhombic net and thus all isotropic dihedral angles of *q* agree (see [32] for the Euclidean counterpart): The parameter lines of the K-net *k* posses constant discrete isotropic torsion; they belong to linear line complexes and the entire net can be generated by so-called isotropic Clifford translation, like their smooth counterparts [13].

Finally, we address the question whether an isotropic V-net can be embedded into a one-parameter family of V-nets that are isometric to each other. In isotropic geometry, the definition of isometry cannot be based only on the metric on the surface, which is seen in the top view. However, we can in addition require that isotropic Gaussian curvature is preserved. As discrete isotropic Gaussian curvature we use the quotient of the area of the face of the Gauss image divided by a weighted area of the corresponding vertex star on the given net (cf. Equation (3)). The following theorem will hold for any definition of wighted area of the vertex star which depends affinely on the area of the vertex star (which is the case for commonly used definitions).

We will report on isometries in isotropic space in a separate publication, but briefly mention the embedding of a V-net *q* into a one-parameter family of isometric V-nets by showing the following result.

#### Theorem 14

An isotropic V-net *q* can be embedded into a continuous family of isometric isotropic V-nets $q(\lambda )$ so that all nets in the family not only have the same top view (i.e., same metric), but also the same isotropic Gaussian curvature in corresponding vertices.

#### Proof

Applying the polarity ${\tilde{\delta }}$ with respect to *S* (see 4), which is also a metric duality in isotropic space $I_3$, Lemma 3 implies that the dual net $q^*$ of *q* has the same top view as the Gaussian image $q_S$ of *q*. The vertices of the net $q_S$ are on *S* and the tangent planes of *S* at them are parallel to the corresponding face planes of *q*.

With the same arguments as in the proof of Theorem 11, the top view $q'_S = q^*{}'$ is a translational net. Two successive parameter lines in $q^*$ are related by a translation. We scale each individual such translation vector by a factor $\lambda$ (inducing a Combescure transformation, cf. Definition 1) and it will still generate a net Q-net with the above properties. Analogously we scale the parameter lines in the other direction with $1/\lambda$ and obtain, after scaling in both directions, a Q-net $q^*(\lambda )$. This Combescure transformation leaves the areas of all parallelograms unchanged. Now we map such a Q-net $q^*(\lambda )$ via ${\tilde{\delta }}$ back to an isotropic V-net. Since all nets $q^*(\lambda )$ have parallel corresponding face planes, all nets $q(\lambda )$ have the same top view (Lemma 3). The isotropic areas (areas of top views) of the Gaussian images $q_S(\lambda )$ of the vertices in $q(\lambda )$ (areas of faces of $q^*(\lambda )$) do not depend on $\lambda$. Consequently, the discrete Gaussian curvature (3) of all nets in the family $q(\lambda )$ is the same. $\square$

Note that the properties of the metric duality (Lemma 1) imply that $\lambda$ and $1/\lambda$ (in the proof above) are the factors with which the dihedral angles of *q* get multiplied to obtain $q(\lambda )$.

### Two versions of infinitesimal flexibility, Kœnigs nets and dual Kœnigs nets

The discrete theory of infinitesimally flexible nets has been studied in detail by Sauer [30]. In fact, most of Sauer’s results concern two special types of infinitesimal flexibility:

(i) *Flexibility with rigid faces*: A net *q* has this type of flexibility if there is a vector field, attached to the vertices of the net, so that per face the vector field belongs to a velocity field of a rigid body motion in Euclidean 3-space. This type of flexibility is equivalent to the existence of a *reciprocal-parallel* net *k* (and thus equivalent to static equilibrium of *q* without external forces applied to inner vertices).
(ii) *Flexibility with rigid vertex stars*: Here, a vector field is attached to the vertices of the net which agrees with the velocity field of a rigid body motion per vertex star. In other words, the edge lines through a vertex form a pyramid, which is infinitesimally rigid. This type of flexibility requires a Q-net *q* and is characterized by the existence of a Q-net ${\hat{q}}$, which is *antiparallel* to *q*. This means that corresponding edges and not corresponding face diagonals are parallel. The existence of an antiparallel net is a known characterization of *Kœnigs nets* (see also [17]).

Sauer shows that each of the two classes of infinitesimally flexible nets is invariant under projective maps [30]. Furthermore, a projective duality (correlation) maps a Q-net of one type to a Q-net of the other type [30]. Hence, Q-nets which are flexible with rigid faces are projective duals of Kœnigs nets. Remarkably, the Q-nets appearing in discrete isotropic AGAG-webs belong to both classes.

#### Theorem 15

The isotropic V-nets *q* contained in a discrete AGAG-web are Kœnigs nets and dual Kœnigs nets.

#### Proof

By Theorem 13 (iii) there exists a reciprocal-parallel net (isotropic K-net *k*) and thus *q* is a dual Kœnigs net. To show that *q* is also a Kœnigs net, we prove that the projectively dual timelike minimal net $q^*$ in Minkowski space is a dual Kœnigs net: Each edge of $q^*$ is isotropic and thus parallel to a ruling of the minimal cone $x_1^2 + x_2^2 - x_3^2 = 0$ and also parallel to a ruling in an A-net *s* on the Minkowski sphere $x_1^2 + x_2^2 - x_3^2 = 1$. The edges in a transversal sequence of $q^*$ (joining neighboring parameter lines) are parallel to each other and thus parallel to a parameter line (ruling) of *s*, showing the reciprocal-parallelism of *s* and $q^*$ and the dual Kœnigs property of $q^*$. $\square$

The proof reveals another analogy between timelike minimal surfaces in Minkowski space and Euclidean minimal surfaces. In both cases, the minimal surface and the sphere carry reciprocal force diagrams, a fact which has been pointed out in the Euclidean case by Blaschke [33].

Since an isotropic V-net *q* is a Kœnigs net there is a Kœnigs dual ${\hat{q}}$ which is antiparallel to *q*. The dual net ${\hat{q}}$ shows the same properties as the given net:

#### Theorem 16

The Kœnigs dual (antiparallel) net ${\hat{q}}$ of an isotropic V-net *q* is also an isotropic V-net. If the geodesics of one net lie in the tangent planes of an isotropic quadratic cylinder, the same is true for the other net.

#### Proof

Planar parameter lines are mapped to planar parameter lines by any discrete Combescure transformation (Definition 1). Hence, an isotropic V-net, characterized by parameter lines in isotropic planes, is mapped to an isotropic V-net by any Combescure transformation. Let us now take a net *q* whose parameter lines lie in the tangent planes of an isotropic quadratic cylinder. In the top view, the parameter lines of $q'$ are straight and the tangents of a conic. The top view ${\hat{q}}'$ of the Kœnigs dual net is also a Kœnigs net whose parameter lines are straight. Theorem 5 implies that this net is the top view of an A-net *a*. As an A-net, it has planar vertex stars, which implies straight parameter lines and thus *a* consists of rulings of a quadric. Hence, in the top view, the parameter lines are tangents of a conic, the contour of the quadric in the top view. $\square$

A geodesic net *q* from a discrete AGAG-web has two diagonal A-nets (cf. the table in the appendix). Therefore its antiparallel (or Kœnigs dual) net ${\hat{q}}$ has diagonal Q-nets and thus is not part of a non-planar isotropic AGAG-web. While *q* represents a surface of negative Gaussian curvature, ${\hat{q}}$ is positively curved. If we view ${\hat{q}}$ as relative sphere then *q* is a relative minimal surface.

### Conclusion and future research

Our problem has been motivated by a special type of gridshells that can be fabricated by bending straight and flat slats. The corresponding curve network must follow the curves of an AGAG-web. The existence of such smooth AGAG-webs in Euclidean space so far has only been verified by numerical methods. While in Euclidean space the problem is formulated between projective geometry (A-nets) and Euclidean geometry (geodesics), in isotropic space the problem becomes purely projective as the geodesics lie in isotropic planes. We could therefore describe all discrete AGAG-webs in isotropic geometry and showed that these planes must be tangent to a quadratic cylinder. The resulting surfaces exhibit relations to timelike minimal surfaces in Minkowski space and to isotropic counterparts of Voss-nets.

The present paper opens up several directions for future research.

(i) The mathematical existence of smooth and discrete Euclidean AGAG-webs remains an open problem. Our results on the isotropic counterpart could serve as initial guesses in optimization algorithms for computing discrete Euclidean AGAG-webs [10].
(ii) In the present paper we discussed 4-webs of asymptotic lines and curves in planes of a bundle. Are there other 4-webs of asymptotic lines and planar curves?
(iii) The Q-nets in a discrete AGAG-web turned out to be Kœnigs and dual Kœnigs nets. It is interesting to determine all Q-nets with that property.
(iv) The newly introduced concept of isotropic isometries between surfaces turns out to yield interesting results on which we will report in a forthcoming publication.

## Appendix

Here we provide a table of the combinatorics of the sub-nets of AGAG-webs which appear in the paper. In the first row we have the base net which is the net of geodesics followed by its two diagonal nets (1st and 2nd A-net). In the second row we illustrate all four possible diagonal nets of the two A-nets. Below the images we list in what form they appear in the paper. The abbreviation **tv** means “top view”.

## Declarations

### Editorial Policies for:

Springer journals and proceedings: https://www.springer.com/gp/editorial-policies Nature Portfolio journals: https://www.nature.com/nature-research/editorial-policies*Scientific Reports*: https://www.nature.com/srep/journal-policies/editorial-policies BMC journals: https://www.biomedcentral.com/getpublished/editorial-policies.
